# County-Level Enrollment in Medicare Advantage Plans Offering Expanded Supplemental Benefits

**DOI:** 10.1001/jamanetworkopen.2024.33972

**Published:** 2024-09-17

**Authors:** Zhiyou Yang, Emily Zhu, David Cheng, Mary Price, Margarita Alegria, John Hsu, Joseph P. Newhouse, Vicki Fung

**Affiliations:** 1 The Mongan Institute, Massachusetts General Hospital, Boston; 2 Department of Health Policy and Management, Harvard T.H. Chan School of Public Health, Boston, Massachusetts; 3 Department of Medicine, Harvard Medical School, Boston, Massachusetts; 4 Biostatistics Center, Massachusetts General Hospital, Boston; 5 Harvard Kennedy School, Cambridge, Massachusetts; 6 National Bureau of Economic Research, Cambridge, Massachusetts; 7 Department of Health Care Policy, Harvard Medical School, Boston, Massachusetts

## Abstract

**Importance:**

Since 2019 and 2020, Medicare Advantage (MA) plans have been able to offer supplemental benefits that address long-term services and supports (LTSS) and social determinants of health (SDOH).

**Objective:**

To examine the temporal trends and geographic variation in enrollment in MA plans offering LTSS and SDOH benefits.

**Design, Setting, and Participants:**

This cross-sectional study used publicly available data to examine changes in beneficiary enrollment and plan offerings of LTSS and SDOH benefits from the benefits data from the second quarter of each year and other data from April of each year except 2024, for which the first quarter was the latest for benefits data and January the latest for other data at the time of analysis. Multivariable linear regression models for each type of benefit were used to investigate associations between county characteristics and enrollment in 2024. Analyses were stratified for (1) Dual Eligible Special Needs Plans (D-SNPs) that exclusively enroll dual-eligible beneficiaries and (2) non–D-SNPs.

**Main Outcomes and Measures:**

The percentage of MA enrollees in plans offering LTSS or SDOH benefits at the county level.

**Results:**

This study included 2 631 697 D-SNP and 20 114 506 non–D-SNP enrollees in 2020, which increased to 5 494 426 and 25 561 455, respectively, in 2024. From 2020 to 2024, the percentage of D-SNP enrollees in plans offering SDOH benefits increased from 9% to 46%, whereas the percentage fluctuated between 23% and 39% for LTSS benefits. There was an increase in non–D-SNP enrollees with LTSS (from 9% to 22%) and SDOH (from 4% to 20%) benefits from 2020 to 2023, which decreased in 2024. In 2024, the most offered LTSS benefit was in-home support services, and the most offered SDOH benefit was food and produce. The percentage of enrollees with these benefits varied across counties in 2024. In multivariable linear regression models, among D-SNPs, enrollment in plans offering any SDOH benefits was higher in counties with greater MA penetration (coefficient, 5.0 percentage points [pp] per 10-pp change; 95% CI, 2.1-7.9 pp), in urban counties (coefficient, 7.2 pp vs rural counties; 95% CI, 3.8-10.6 pp), in counties with greater enrollment in fully integrated D-SNPs (coefficient, 3.0 pp per 10-pp change; 95% CI, 2.2-3.9 pp), and in counties in states with approved Medicaid home- and community-based services waivers for individuals 65 years or older or those with disabilities (coefficient, 10.8 pp; 95% CI, 4.0-17.6 pp). Enrollment in D-SNPs offering LTSS benefits was also higher in counties with greater MA penetration (coefficient, 5.9 pp per 10-pp change; 95% CI, 2.4-9.5 pp), urban vs rural counties (coefficient, 4.6 pp; 95% CI, 1.1-8.1 pp), and counties with greater enrollment in fully integrated D-SNPs (coefficient, 3.0 pp per 10-pp change; 95% CI, 2.1-3.9 pp) in addition to counties with greater social vulnerability scores (coefficient, 1.4 pp per 10-pp change; 95% CI, 0.3-2.5 pp).

**Conclusions and Relevance:**

In this cross-sectional study of MA plans and enrollees, an increase in enrollment was most consistent in D-SNPs offering SDOH benefits compared with LTSS benefits and in D-SNPs compared with non–D-SNPs. Geographic variation in enrollment patterns highlights potential gaps in access to LTSS and SDOH benefits for rural MA beneficiaries and dual-eligible enrollees living in counties with lower enrollment in fully integrated D-SNPs and states with more limited Medicaid home- and community-based services coverage.

## Key Points

**Question:**

What are the enrollment patterns in Medicare Advantage (MA) plans offering supplemental long-term services and supports (LTSS) and social determinants of health (SDOH) benefits?

**Findings:**

In this cross-sectional study of MA plan enrollees, an increase in enrollment was most consistent in Dual Eligible Special Needs Plans (D-SNPs) offering SDOH vs LTSS benefits and in D-SNPs vs non–D-SNPs from 2020 (483 D-SNPs with 2 631 697 enrollees and 3207 non–D-SNPs with 20 114 506 enrollees) to 2024 (786 D-SNPs with 5 494 426 enrollees and 4143 non–D-SNPs with 25 561 455 enrollees). In 2024, enrollment in plans with these benefits was higher in urban counties, counties with greater MA penetration and fully integrated D-SNP enrollment, and states with Medicaid home- and community-based services waivers.

**Meaning:**

Increasing enrollment in MA plans offering LTSS or SDOH benefits may improve access to these services, but geographic variation remains.

## Teaser

This cross-sectional study examines trends in enrollment in Medicare Advantage plans that offered long-term services and supports and social determinants of health benefits from 2020 to 2024.

## Introduction

An increasing number of Medicare beneficiaries are electing to receive their Medicare benefits through private Medicare Advantage (MA) plans. In 2022, 45% of all Medicare beneficiaries and 56% of dually eligible Medicare-Medicaid beneficiaries were enrolled in MA plans, increasing from 39% and 45%, respectively, from 2020.^1^ Medicare Advantage plans often provide supplemental benefits not covered by traditional Medicare, such as dental, vision, and hearing care. Recently, the Centers for Medicare & Medicaid Services (CMS) authorized additional flexibility for MA plans to offer supplemental benefits that address needs related to long-term services and supports (LTSS) and social determinants of health (SDOH).

There are several authorities through which MA plans can offer these benefits. Starting in 2019, the CMS expanded its definition of primarily health-related MA supplemental benefits to include LTSS-type services, such as adult day care services, in-home support services, and support for caregivers of enrollees (eg, respite care).^2^ Starting in 2020, MA plans were authorized to offer special supplemental benefits for the chronically ill (SSBCI) that cover non–primarily health-related benefits to address SDOH, such as food and produce, nonmedical transportation, and pest control, in addition to primarily health-related benefits, to beneficiaries with certain chronic conditions.^3^ Plans can also offer these supplemental benefits through 2 other authorities: the value-based insurance design (VBID) model and uniformity flexibility (UF).^4^ The VBID model also allows plans to offer benefits to beneficiaries based on socioeconomic status in addition to chronic conditions.^4^ Participation in the VBID model by MA plans has increased substantially since its launch in 2017, although as of 2022, most SDOH benefits were provided through SSBCI.^4,5^

In 2022, approximately 19% of MA plans offered LTSS, and 21% offered SDOH benefits, varying by plan type and county; in contrast, approximately 11% and 6% offered them, respectively, in 2020.^4^ Prior work^6^ has also found greater availability of LTSS benefits in urban vs rural counties, counties with higher social vulnerabilities, and counties with higher MA penetration. In addition, SDOH benefits were more commonly offered by Dual Eligible Special Needs Plans (D-SNPs) that exclusively enroll dually eligible Medicare-Medicaid beneficiaries compared with non–D-SNPs.^7^ Although these LTSS and SDOH benefits in MA plans could provide greater access to needed services, potential overlap in services covered by MA plans and state Medicaid programs could contribute to coordination challenges for dually eligible Medicare-Medicaid beneficiaries.^8^ This potential overlap is more likely in states with approved Medicaid waivers for home- and community-based services (HCBS). However, there is limited evidence on how MA enrollment patterns in plans offering LTSS or SDOH benefits vary across states with and without HCBS waivers. This study aims to examine these geographic patterns using the most recent data available given the ongoing increase in MA enrollment and changes in MA plan offerings.

## Methods

### Data Sources and Study Population

In this cross-sectional study, we used publicly available CMS MA plan data, including benefits and enrollment, to examine trends in MA beneficiaries’ enrollment in plans that offered recently authorized LTSS and SDOH benefits and MA plans’ offerings of these benefits from 2020 to 2024 for D-SNPs vs non–D-SNPs. We then examined associations between county and state characteristics and county-level enrollment or plan offerings of these benefits in 2024. Information on enrollees’ race and ethnicity was not available in the data. The Mass General Brigham Institutional Review Board determined that this project meets criteria for secondary research for which consent is not required. The study followed the Strengthening the Reporting of Observational Studies in Epidemiology (STROBE) reporting guideline for cross-sectional studies.

We used publicly available CMS MA plan data on benefits, enrollment, service area, and D-SNP status as well as county-level MA penetration (ie, the percentage of all Medicare beneficiaries enrolled in MA by county).^9^ We used benefits data from the second quarter of each year and other data from April of each year except 2024, for which the first quarter was the latest for benefits data and January the latest for other data at the time of analysis. We used 2013 Rural-Urban Continuum Codes from the US Department of Agriculture and 2020 Social Vulnerability Index (SVI) data from the Centers for Disease Control and Prevention and Agency for Toxic Substances and Disease Registry, both most recent at the time of analysis.^10,11^ We summarized each state’s Medicaid HCBS waiver availability for individuals 65 years or older or with disabilities based on information on approved waivers from CMS as of April 2023.^12^

Our analysis focused on the MA plan types that offer both D-SNPs and non–D-SNPs, including health maintenance organization (HMO), HMO point of service, and local and regional preferred provider organization plans, which represented 93% of all MA plans in the 2024 data. We excluded Medicare-Medicaid HMO and HMO point of service plans, which are required to cover all Medicare and Medicaid benefits for dually eligible Medicare-Medicaid beneficiaries, and other special needs plans (ie, chronic condition and institutional special needs plans), which limit enrollment to special populations and represented approximately 2% of MA enrollment in 2023.^13^ We also restricted our analysis to the 50 states and Washington, DC.

### Statistical Analysis

#### Analysis of Trends in Enrollment and Plan Offerings

We describe the annual percentage of MA enrollees in plans offering LTSS or SDOH benefits and the percentage of MA plans offering these benefits between 2020 and 2024 separately for D-SNPs and non–D-SNPs. We further examined enrollment and plan offerings for each LTSS and SDOH benefit type as specified in the CMS MA benefits data. Lastly, we examined trends in the percentage of plans that offered these benefits via expanded primarily health-related benefits, SSBCI, VBID, or UF authorities each year.

#### Analysis of Geographic Variation

We assessed the percentage of MA enrollees in plans offering LTSS or SDOH benefits and the percentage of MA plans offering these benefits at the county level in 2024 stratified by D-SNPs and non–D-SNPs. We used multivariable linear regression models to examine county-level characteristics associated with these outcomes in 2024. Explanatory variables included county-level MA penetration, urban vs rural status (urban counties were those with US Department of Agricultural Rural-Urban Continuum Codes of 1-4 and rural counties were those with codes of 5-9), and the overall SVI percentile ranking. The Centers for Disease Control and Prevention and Agency for Toxic Substances and Disease Registry SVI was created using area-level information in the US Census American Community Survey (5-year estimates) to reflect 4 demographic and socioeconomic themes (ie, socioeconomic status, household characteristics, racial and ethnic minority status, and housing type and transportation) related to a community’s vulnerability to natural and human-caused disasters.^14^

We also examined whether states had any approved Medicaid HCBS waivers for individuals 65 years or older or with disabilities because there are potential similarities in the benefits offered under the LTSS and SDOH benefits through MA plans and services covered by Medicaid HCBS waivers for dual-eligible enrollees (eg, adult day health services, general supports for living including housing supports) (eFigure 1 in Supplement 1).^15^ We excluded waivers that specified different target populations, such as individuals with intellectual or developmental disabilities, autism, or HIV and/or AIDS. For analysis of D-SNPs, we also adjusted for the county-level percentage of D-SNP enrollees in fully integrated D-SNPs, which provide Medicare and Medicaid benefits under a single legal entity and satisfy additional integration requirements, such as coverage of both LTSS and behavioral health services in their Medicaid contracts with states.^16,17^

To improve the representativeness of our estimates and account for varying precision in county-level estimates, we weighted the models by county-level numbers of D-SNP or non–D-SNP enrollees or plans (depending on the outcome). We used robust SEs. Coefficients for explanatory variables measured in percentages (ie, MA penetration, SVI percentile rank, and percentage of D-SNP enrollees in fully integrated SNPs) represent the associations between the outcome and per 10–percentage point change (for ease of interpretation) in the explanatory variables. We used Stata, version 18 (StataCorp LLC) for statistical analysis.

## Results

### Trends in Enrollment and Plan Offerings of LTSS or SDOH Benefits

In 2020, 483 D-SNPs (with 2 631 697 enrollees) and 3207 non–D-SNPs (with 20 114 506 enrollees) were included in the study, which increased to 786 D-SNPs (with 5 494 426 enrollees) and 4143 non–D-SNPs (with 25 561 455 enrollees) in 2024. Among all MA plans in 2020, 464 of 3690 plans (13%) offered either of the LTSS or SDOH benefits, comprising 2 872 726 of 22 746 203 enrollees (13%) vs 1739 of 4929 plans (35%) and 10 012 755 of 31 055 881 enrollees (32%) in 2024.

**Figure 1.**
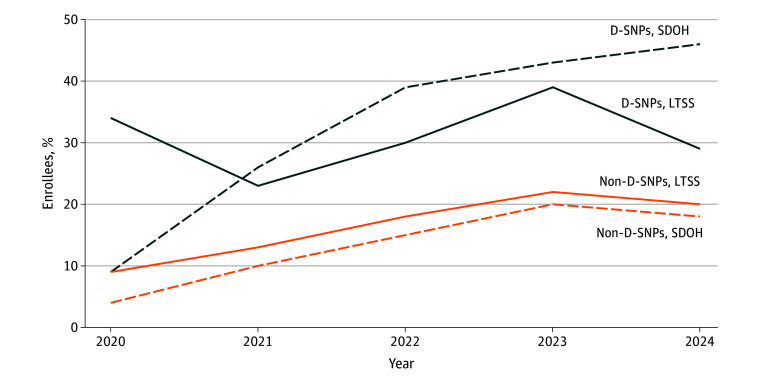
Percentage of Medicare Advantage Enrollees in Plans Offering Long-Term Services and Supports (LTSS) or Social Determinants of Health (SDOH) Benefits, 2020-2024 D-SNP indicates Dual Eligible Special Needs Plan.

Plan offerings and enrollment varied by benefit and plan types. For example, from 2020 to 2024, the percentage of enrollees in MA plans that offered any LTSS benefits varied between 23% (2021) and 39% (2023) among D-SNPs and increased from 9% in 2020 to 22% in 2023 and then decreased to 20% in 2024 among non–D-SNPs. The percentage of enrollees in D-SNPs with any SDOH benefits increased each year between 2020 and 2024 from 9% to 46%; among non–D-SNP enrollees, this percentage increased from 4% to 20% in 2020 to 2023 and then decreased to 18% in 2024 (Figure 1; eTable 1 in Supplement 1). The percentage of MA plans offering these benefits followed similar patterns; for example, among D-SNPs, 7% provided SDOH benefits in 2020, which increased to 70% in 2024 (eTable 2 in Supplement 1).

### Authorities Used by Plans to Offer LTSS or SDOH Benefits

Between 2020 and 2024, there were shifts in the authorities used by plans to offer these benefits, with a greater share of D-SNPs using the VBID authority over time to provide both LTSS and SDOH benefits. For example, among D-SNPs offering SDOH benefits, all used the SSBCI authority in 2020; in 2024, 65% offered SDOH benefits solely through the VBID model, and 26% solely used SSBCI (9% of D-SNPs used multiple authorities) (eFigure 2 in Supplement 1). Among non–D-SNPs, which are less likely to participate in the VBID model, most SDOH benefits were provided through the SSBCI authority across all years; LTSS benefits were offered primarily through expanded primarily health-related benefits and SSBCI.

### Types of LTSS or SDOH Benefits Offered

**Figure 2.**
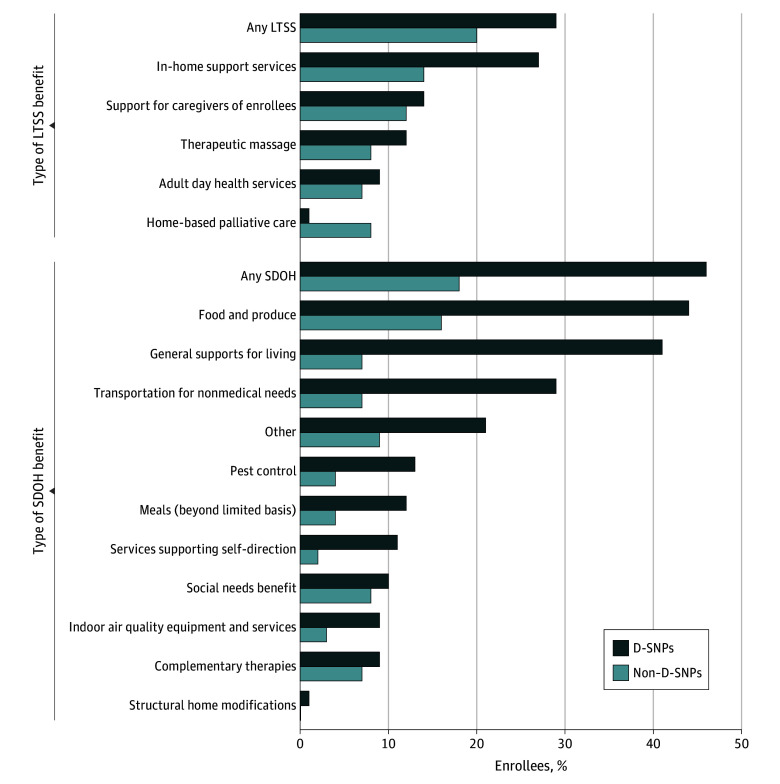
Percentage of Medicare Advantage Enrollees in Plans Offering Each Type of Long-Term Services and Supports (LTSS) or Social Determinants of Health (SDOH) Benefit, 2024 D-SNP indicates Dual Eligible Special Needs Plan.

The availability of different types of LTSS and SDOH benefits varied widely in 2024 (Figure 2). For LTSS benefits, enrollees were most commonly in plans offering in-home support services (27% of D-SNP and 14% of non–D-SNP enrollees) followed by support for caregivers of enrollees (14% of D-SNP and 12% of non–D-SNP enrollees). In contrast, only 1% and 8% of D-SNP and non–D-SNP enrollees were in plans offering home-based palliative care. For SDOH benefits, enrollees were most commonly in plans that offered food and produce benefits (44% of D-SNP and 16% of non–D-SNP enrollees) and general supports for living (which includes subsidies for rent or utilities and assisted living [41% for D-SNP and 7% of non–D-SNP enrollees]). Meanwhile, 1% and 0% of D-SNP and non–D-SNP enrollees were in plans offering structural home modifications. Medicare Advantage plan offerings mirrored the enrollment trends (eTable 2 in Supplement 1). The decreases in enrollment and plan offerings of LTSS benefits from 2023 to 2024 were primarily driven by decreases in in-home support services (eTables 1 and 2 in Supplement 1).

### Geographic Variation in Enrollment and Plan Offerings of LTSS or SDOH Benefits

**Figure 3.**
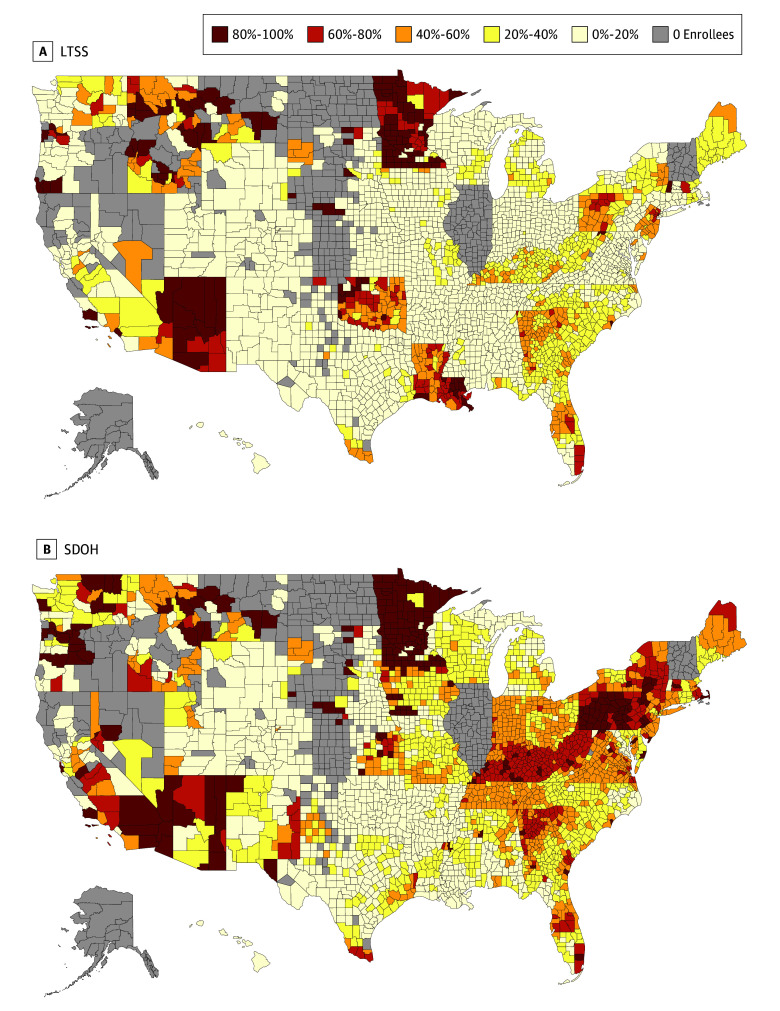
Percentage of Dual Eligible Special Needs Plan (D-SNP) Enrollees in Plans Offering Long-Term Services and Supports (LTSS) or Social Determinants of Health (SDOH) Benefits by County, 2024 The median (IQR) percentage of D-SNP enrollees at the county level enrolled in plans offering LTSS benefits was 14% (0%-33%), and for SDOH benefits it was 36% (16%-55%). Similar maps for non–D-SNP enrollees are available in eFigure 3 in Supplement 1.

There was substantial geographic variation in the percentage of MA enrollees in plans with any LTSS or SDOH benefits at the county level in 2024 among both D-SNPs and non–D-SNPs. The median (IQR) percentage of D-SNP enrollees at the county level enrolled in plans offering LTSS benefits was 14% (0%-33%) and for SDOH benefits was 36% (16%-55%) (Figure 3). Among non–D-SNP enrollees, the median (IQR) percentage at the county level in plans offering LTSS benefits was 10% (2%-22%) and for SDOH benefits was 12% (4%-27%) (eFigure 3 in Supplement 1).

In multivariable linear regression models (Table), among D-SNPs, enrollment in plans offering any SDOH benefits was higher in counties with greater MA penetration (coefficient, 5.0 percentage points [pp] per 10-pp change; 95% CI, 2.1-7.9 pp), in urban counties (coefficient, 7.2 pp vs rural counties; 95% CI, 3.8-10.6 pp), in counties with greater enrollment in fully integrated D-SNPs (coefficient, 3.0 pp per 10-pp change; 95% CI, 2.2-3.9 pp), and in counties in states with approved Medicaid HCBS waivers for individuals 65 years or older or those with disabilities (coefficient, 10.8 pp; 95% CI, 4.0-17.6 pp). Enrollment in D-SNPs offering LTSS benefits was also higher in counties with greater MA penetration (coefficient, 5.9 pp per 10-pp change; 95% CI, 2.4-9.5 pp), urban vs rural counties (coefficient, 4.6 pp; 95% CI, 1.1-8.1 pp), and counties with greater enrollment in fully integrated D-SNPs (coefficient, 3.0 pp per 10-pp change; 95% CI, 2.1-3.9 pp). Enrollment was also higher in counties with greater social vulnerability scores (coefficient, 1.4 pp per 10-pp change; 95% CI, 0.3-2.5 pp) but not higher in states with the Medicaid HCBS waivers.

**Table.** Association Between Area Characteristics and County-Level Percentage of MA Enrollees in Plans Offering LTSS or SDOH Benefits in 2024^a^

Characteristic	Value	Coefficient (95% CI), pp	
		LTSS	SDOH
**Counties with D-SNPs (n = 2713)**			
County MA penetration % (per 10-pp change), mean (SD)	46.4 (13.8)	5.9 (2.4 to 9.5)	5.0 (2.1 to 7.9)
County urban (vs rural) status, No. (%)	1294 (47.7)	4.6 (1.1 to 8.1)	7.2 (3.8 to 10.6)
County Social Vulnerability Index percentile (per 10-pp change), mean (SD)	52.5 (28.4)	1.4 (0.3 to 2.5)	−0.1 (−1.2 to 1.0)
County fully integrated D-SNP enrollment % (per 10-pp change), mean (SD)	7.4 (21.8)	3.0 (2.1 to 3.9)	3.0 (2.2 to 3.9)
Approved state Medicaid home- and community-based services waivers for individuals aged ≥65 y or with disabilities (vs not), No. (%)	2251 (83.0)	2.2 (−7.8 to 12.2)	10.8 (4.0 to 17.6)
**Counties with non–D-SNPs (n = 3047)**			
County MA penetration % (per 10-pp change), mean (SD)	44.2 (15.1)	1.5 (0.4 to 2.5)	0.0 (−1.3 to 1.3)
County urban (vs rural) status, No. (%)	1378 (45.2)	5.4 (3.7 to 7.1)	−0.3 (−2.1 to 1.5)
County Social Vulnerability Index percentile (per 10-pp change), mean (SD)	50.2 (28.7)	−0.4 (−0.9 to 0.0)	−0.3 (−0.9 to 0.2)
Approved state Medicaid home- and community-based services waivers for individuals aged ≥65 y or with disabilities (vs not), No. (%)	2552 (83.8)	4.1 (1.1 to 7.1)	5.4 (2.5 to 8.3)

Abbreviations: D-SNP, Dual Eligible Special Needs Plan; LTSS, long-term services and supports; MA, Medicare Advantage; pp, percentage points; SDOH, social determinants of health.

^a^ This table presents the results of multivariable linear regression models examining the association between area characteristics and enrollment in 2024, weighted by county-level numbers of D-SNP or non–D-SNP enrollees, with robust SEs.

Among non–D-SNPs, enrollment in plans offering SDOH benefits was higher in states with approved HCBS waivers for individuals 65 years or older or with disabilities (coefficient, 5.4 pp; 95% CI, 2.5-8.3 pp); enrollment in plans offering LTSS benefits was higher in counties with greater MA penetration (coefficient, 1.5 pp per 10-pp change; 95% CI, 0.4-2.5 pp), in urban counties (coefficient, 5.4 pp; 95% CI, 3.7-7.1 pp), and in counties with the Medicaid HCBS waivers (coefficient, 4.1 pp; 95% CI, 1.1-7.1). Associations between the percentage of plans offering these benefits and area characteristics were generally similar to enrollment patterns (eTable 3 in Supplement 1).

## Discussion

We found that enrollment in and plan offerings of recently authorized SDOH supplemental benefits increased substantially between 2020 and 2024 among D-SNPs. Nearly half of D-SNP enrollees were in plans offering SDOH benefits in 2024. In contrast, D-SNP enrollment and plan offerings of LTSS benefits fluctuated during the same period. In addition, enrollment in non–D-SNPs offering LTSS or SDOH benefits was comparatively lower than in D-SNPs, and the increasing trend in enrollment between 2020 and 2023 decreased in 2024.

Although use of these benefits remains unknown, it is possible that fluctuations over time in MA enrollment in plans that provide LTSS benefits reflect challenges associated with providing these benefits, such as workforce shortages that were exacerbated by the COVID-19 pandemic.^18^ In fact, reductions in enrollment in D-SNPs offering LTSS benefits between 2023 and 2024 were primarily driven by decreases in the share of plans offering in-home support services, which include provision of personal care assistance to beneficiaries to assist with activities of daily living.^19^ In contrast, D-SNP enrollment in plans offering SDOH benefits increased substantially during the study period. The most offered SDOH benefits in 2024 were food and produce (eg, grocery gift cards) and general supports for living (eg, subsidies for rent or utilities), which could be easier to implement compared with other types of services.

An increasing share of D-SNPs over time used the VBID model to offer LTSS and SDOH benefits, which has been extended through 2030.^20^ In addition to the flexibilities under this model to provide these additional supplemental benefits, plans participating in the VBID model are also able to reduce cost-sharing for Part D drugs and, starting in 2025, for certain Part C services.^20^ In addition, in contrast to the SSBCI authority, which only allows plans to target SDOH and LTSS benefits to individuals with chronic conditions, the VBID plans can provide these benefits based on both chronic conditions and socioeconomic status, allowing for potentially broader access across enrollees.

We found geographic variation in enrollment patterns in 2024, with beneficiaries in rural counties less likely to be enrolled in plans offering these benefits. These findings are consistent with work^6^ from prior years that found similar differences, suggesting that rural-urban gaps have remained over time. More limited availability of such benefits in rural areas could reflect previously identified challenges of providing community-based LTSS in rural locations, such as greater workforce shortages, transportation limitations, and telecommunications barriers, compared with urban areas^21,22^; similar barriers to providing services to address SDOH in rural areas have also been identified.^23^ At the same time, evidence finds that rural counties fare worse on SDOH measures than urban counties, on average, and that such gaps have increased over time,^24^ underscoring the urgent need to identify models for delivering these services in rural areas.

Medicare Advantage enrollees living in states with approved Medicaid HCBS waivers for individuals 65 years or older or with disabilities were also more likely to be enrolled in D-SNPs offering SDOH benefits and non–D-SNPs offering LTSS or SDOH benefits. Although dually eligible Medicare-Medicaid beneficiaries may be able to access certain LTSS and SDOH benefits through their Medicaid coverage, the availability of Medicaid HCBS coverage varies substantially, depending on approved services, eligibility criteria, and local capacity.^12,25^ Thus, coverage of these services through MA plans may be an important source of coverage for many dually eligible Medicare-Medicaid beneficiaries, even those in states with coverage of HCBS services. Of concern, however, a report^8^ from the Office of the Assistant Secretary for Planning and Evaluation noted that the overlap between the recently authorized MA benefits and existing Medicaid coverage could create confusion at the beneficiary, plan, and even state levels about whether these benefits are covered by Medicaid or MA plans. State and federal policies aimed at increasing the level of Medicaid-Medicare integration in MA plans for dually eligible Medicare-Medicaid beneficiaries, such as through fully integrated D-SNPs, may help streamline the provision of these benefits between the 2 programs.^17^ Indeed, we found that enrollment in plans offering these benefits was greater among D-SNP enrollees in counties with a greater share of enrollees in fully integrated D-SNPs. Although the percentage of dually eligible Medicare-Medicaid beneficiaries in integrated D-SNPs is increasing over time, most D-SNPs are not fully integrated, and a large share of dually eligible Medicare-Medicaid beneficiaries in MA are enrolled in non–D-SNPs (nearly 40% in 2020),^26^ which have no requirements for coordinating with state Medicaid programs. Thus, attention is needed to the coordination of coverage for these services between MA plans and state Medicaid programs for both D-SNPs and non–D-SNPs.

### Limitations

Our study has several limitations. First, details on the benefits provided by MA plans within each category and the plans’ eligibility criteria for receiving each benefit were limited. Plans can elect to offer these benefits to subsets of enrollees, such as those with certain chronic conditions, and on a limited basis. For example, in 2020, most meal benefits offered by SNPs were limited to 30 days or fewer.^27^ We did not assess variation in the duration or generosity of the benefits provided across plans, which could impact their ability to address beneficiaries’ LTSS and SDOH needs. Second, we defined MA plans based on unique contract-plan identification. Our analysis did not consider plan segments, in which each segment includes different counties, and benefits may vary across segments even in the same plan. However, the number of segmented plans is limited; in 2023, approximately 11% of MA plans were segmented (6% for D-SNPs).^28^ Third, this study used aggregate county-level MA enrollment data. Thus, we did not examine whether MA plan characteristics, such as whether the plan was new or existing, the plan’s other benefits offerings, or quality ratings, were associated with the offerings of the LTSS or SDOH benefits or enrollment. We did not investigate whether MA plans’ offerings of these benefits were associated with increase in MA enrollment. We also did not examine whether beneficiaries chose plans based on their LTSS or SDOH needs. Future research is needed to investigate the individual-level use of these benefits and evaluate the potential value of these benefits for beneficiaries as measured by health outcomes. Previous work^29^ suggests that the use of supplemental benefits offered by MA plans could be limited; however, data on the use of supplemental benefits are currently lacking in MA encounter data.^30^ Urgent efforts are needed to address the barriers to plans reporting utilization information for these services to address these gaps in the evidence.^30^

## Conclusions

In this cross-sectional study of MA supplemental benefits addressing LTSS and SDOH needs between 2020 and 2024, we found a substantial increase in enrollment in D-SNPs offering SDOH benefits but fluctuating trends in LTSS benefits. Among non–D-SNPs, increases in enrollment in plans with these benefits slowed in 2024. We also found geographic variation in MA enrollment patterns in 2024, highlighting potential gaps in access to these benefits, particularly for rural beneficiaries, and potential challenges and opportunities for coordinating these benefits for dual-eligible enrollees.

## Notes

Supplement 1. **eFigure 1.** Number of states offering benefits comparable to MA LTSS or SDOH benefits via Medicaid HCBS waivers for individuals ages 65+ or with disabilities, as of April 2023 **eFigure 2.** Composition of authorities used by MA plans to offer LTSS or SDOH benefits, 2020-2024 **eFigure 3.** Percentage of non-D-SNP enrollees in plans offering LTSS or SDOH benefits, 2024 **eTable 1.** Percentage of MA enrollees in plans offering LTSS or SDOH benefits, 2020-2024 **eTable 2.** Percentage of MA plans offering LTSS or SDOH benefits, 2020-2024 **eTable 3.** Association between area characteristics and county-level percentage of MA plans offering LTSS or SDOH benefits, 2024

Supplement 2.
Data Sharing Statement
