# Supplementary material for: County-Level Enrollment in Medicare Advantage Plans Offering Expanded Supplemental Benefits
Source: JAMA Netw Open. 2024 Sep 17;7(9):e2433972. doi: 10.1001/jamanetworkopen.2024.33972 (PMC11409149; doi:10.1001/jamanetworkopen.2024.33972)
Supplement: Supplement 1. — eFigure 1. Number of states offering benefits comparable to MA LTSS or SDOH benefits via Medicaid HCBS waivers for individuals ages 65+ or with disabilities, as of April 2023 eFigure 2. Composition of authorities used by MA plans to offer LTSS or SDOH benefits, 2020-2024 eFigure 3. Percentage of non-D-SNP enrollees in plans offering LTSS or SDOH benefits, 2024 eTable 1. Percentage of MA enrollees in plans offering LTSS or SDOH benefits, 2020-2024 eTable 2. Percentage of MA plans offering LTSS or SDOH benefits, 2020-2024 eTable 3. Association between area characteristics and county-level percentage of MA plans offering LTSS or SDOH benefits, 2024 [file jamanetwopen-e2433972-s001.pdf]

Supplementary Online Content

Yang Z, Zhu E, Cheng D, et al. County-level enrollment in Medicare Advantage plans offering expanded supplemental benefits. *JAMA Netw Open*. 2024;7(9):e2433972. doi:10.1001/jamanetworkopen.2024.33972

eFigure 1. Number of states offering benefits comparable to MA LTSS or SDOH benefits via Medicaid HCBS waivers for individuals ages 65+ or with disabilities, as of April 2023
eFigure 2. Composition of authorities used by MA plans to offer LTSS or SDOH benefits, 2020-2024
eFigure 3. Percentage of non–D-SNP enrollees in plans offering LTSS or SDOH benefits, 2024
eTable 1. Percentage of MA enrollees in plans offering LTSS or SDOH benefits, 2020-2024
eTable 2. Percentage of MA plans offering LTSS or SDOH benefits, 2020-2024
eTable 3. Association between area characteristics and county-level percentage of MA plans offering LTSS or SDOH benefits, 2024

This supplementary material has been provided by the authors to give readers additional information about their work.

© 2024 Yang Z et al. *JAMA Network Open.*

eFigure 1. Number of states offering benefits comparable to MA LTSS or SDOH benefits via Medicaid HCBS waivers for individuals ages 65+ or with disabilities, as of April 2023

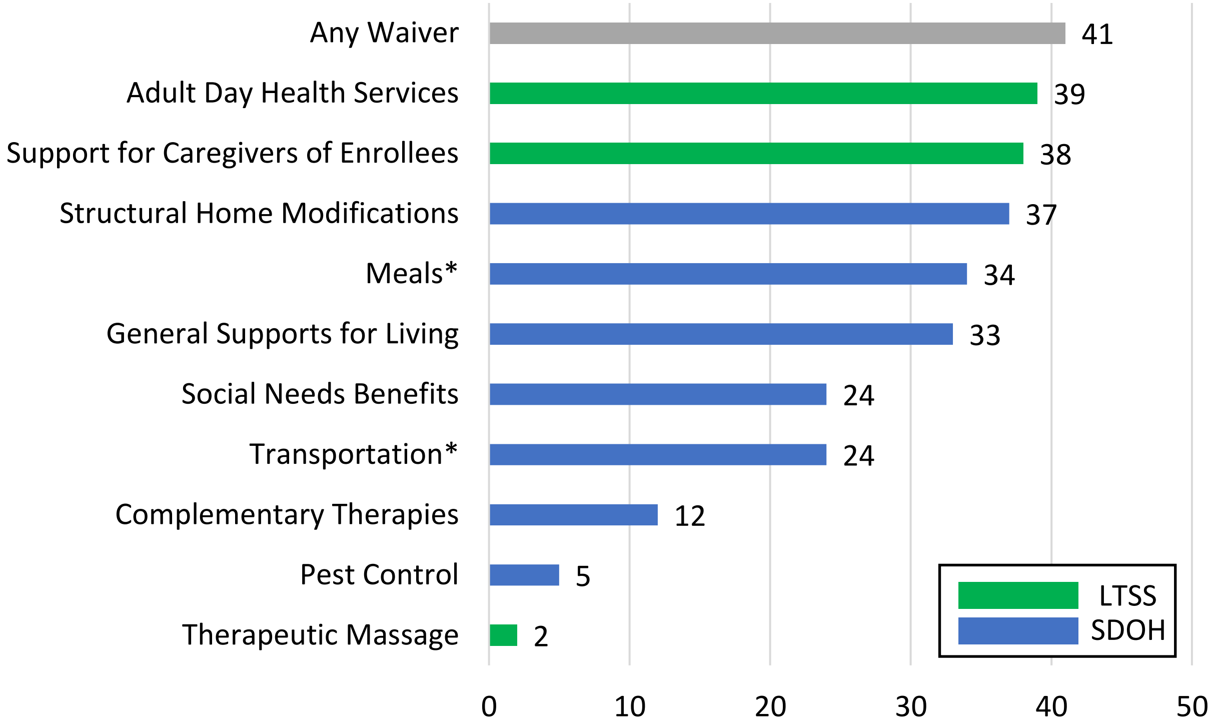

Notes: (1) The MA SDOH benefit of meals is defined as "beyond limited basis" (i.e., beyond the scope of a limited period immediately following a surgery or hospitalization or due to a chronic illness), while the scopes of benefits of meals in Medicaid HCBS waivers are not always clear. (2) The MA SDOH benefit of transportation is for non-medical needs, while the transportation benefits provided via Medicaid HCBS waivers are not always clear in terms of medical vs. non-medical. (3) The MA LTSS benefit of in-home support services is comparable to personal care services provided optionally by Medicaid outside the HCBS waivers (https://www.kff.org/medicaid/report/medicaid-and-long-term-services-and-supports-a-primer/); in 2023, 34 states offered this benefit (https://www.kff.org/medicaid/issue-brief/payment-rates-for-medicaid-home-and-community-based-services-states-responses-to-workforce-challenges/).

© 2024 Yang Z et al. *JAMA Network Open.*

eFigure 2. Composition of authorities used by MA plans to offer LTSS or SDOH benefits, 2020-2024

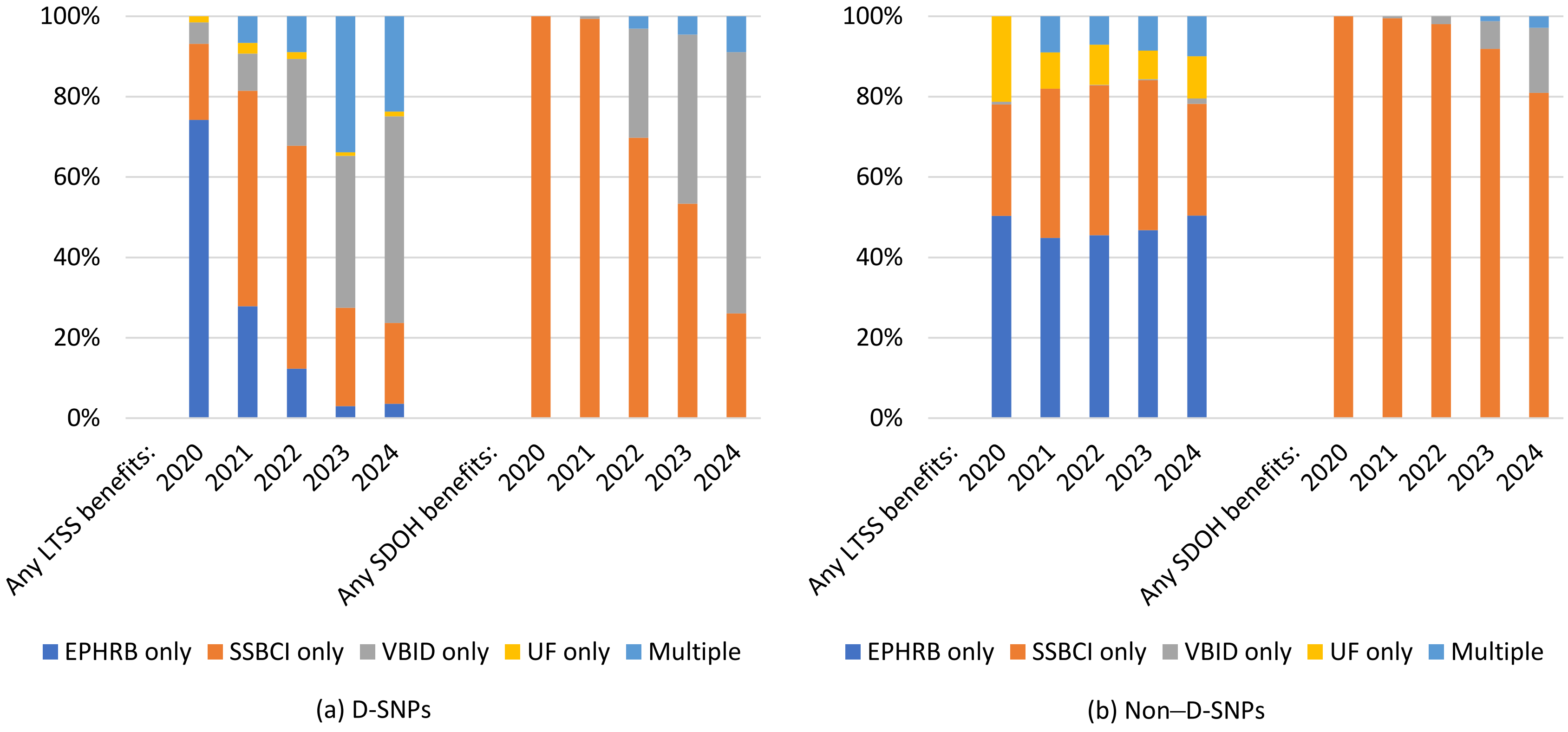

© 2024 Yang Z et al. *JAMA Network Open.*

eFigure 3. Percentage of non–D-SNP enrollees in plans offering LTSS or SDOH benefits, 2024

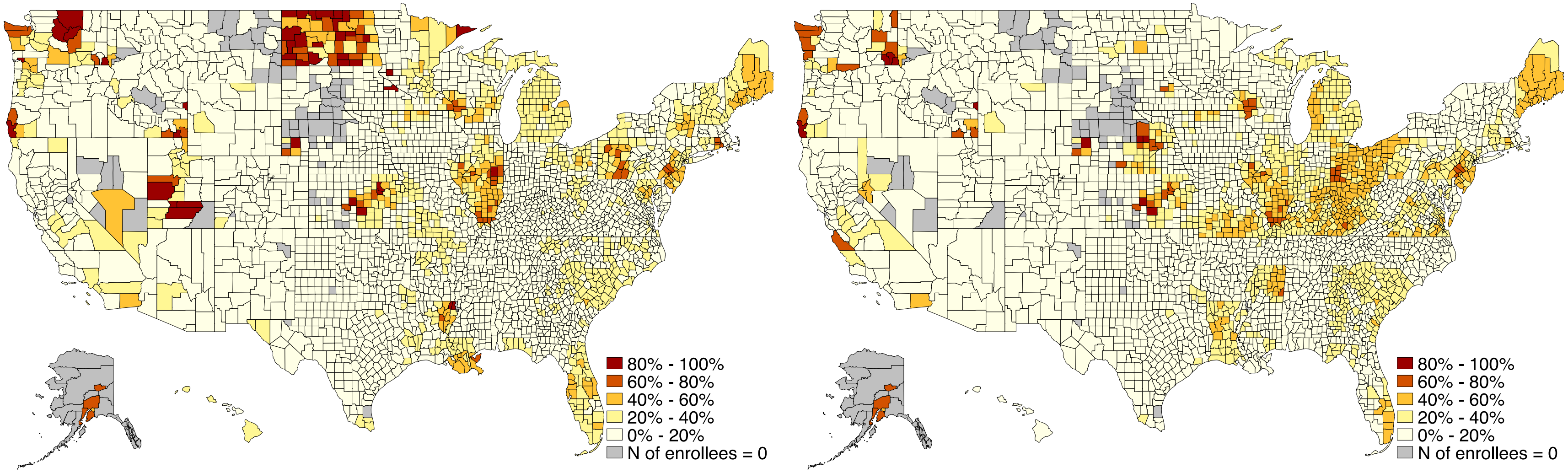

(a) LTSS (b) SDOH

Notes: These maps display the percentage of non–D-SNP enrollees by county in non–D-SNPs that offer (a) LTSS or (b) SDOH benefits in 2024. The median percentage (25th and 75th percentiles) of non–D-SNP enrollees at the county level enrolled in plans offering LTSS benefits was 10% (2%, 22%) and for SDOH benefits was 12% (4%, 27%).

© 2024 Yang Z et al. *JAMA Network Open.*

eTable 1. Percentage of MA enrollees in plans offering LTSS or SDOH benefits, 2020-2024

	D-SNPs					Non–D-SNPs				
Year	2020	2021	2022	2023	2024	2020	2021	2022	2023	2024
N of enrollees	2,631,697	3,149,274	3,895,112	4,945,473	5,494,426	20,114,506	22,009,406	23,427,602	24,679,013	25,561,455
LTSS benefits:										
1 Therapeutic Massage	6%	6%	10%	12%	12%	3%	3%	4%	6%	8%
2 Adult Day Health Services	5%	6%	9%	10%	9%	2%	2%	2%	4%	7%
3 Home-Based Palliative Care	1%	1%	1%	1%	1%	2%	3%	3%	3%	8%
4 In-Home Support Services	13%	17%	27%	36%	27%	4%	6%	13%	17%	14%
5 Support for Caregivers of Enrollees	20%	3%	11%	15%	14%	2%	3%	5%	9%	12%
**Any LTSS benefits**	34%	23%	30%	39%	29%	9%	13%	18%	22%	20%
SDOH benefits:										
1 General Supports for Living	0%	5%	13%	28%	41%	1%	2%	3%	9%	7%
2 Food and Produce	5%	17%	36%	38%	44%	3%	5%	12%	17%	16%
3 Meals (beyond limited basis)	2%	10%	17%	14%	12%	1%	5%	6%	6%	4%
4 Pest Control	5%	11%	16%	13%	13%	3%	4%	5%	6%	4%
5 Transportation for Non-Medical Needs	3%	10%	20%	26%	29%	1%	2%	5%	8%	7%
6 Indoor Air Quality Equipment and Services	2%	8%	10%	11%	9%	1%	2%	2%	6%	3%
7 Social Needs Benefit	1%	6%	4%	13%	10%	1%	3%	7%	11%	8%
8 Complementary Therapies	0%	0%	8%	10%	9%	0%	0%	7%	9%	7%
9 Services Supporting Self-Direction	0%	5%	8%	10%	11%	1%	2%	2%	4%	2%
10 Structural Home Modifications	0%	0%	1%	2%	1%	0%	0%	0%	0%	0%
11 Other	5%	6%	17%	21%	21%	2%	3%	10%	12%	9%
**Any SDOH benefits**	9%	26%	39%	43%	46%	4%	10%	15%	20%	18%
Overall summary:										
Any LTSS or SDOH benefits	36%	34%	45%	51%	53%	10%	16%	26%	31%	28%
Any LTSS but no SDOH benefits	28%	8%	6%	8%	7%	5%	6%	10%	11%	9%
Any SDOH but no LTSS benefits	3%	10%	15%	12%	24%	1%	3%	8%	9%	7%
Both LTSS and SDOH benefits	6%	16%	24%	31%	22%	3%	6%	7%	11%	11%
No LTSS or SDOH benefits	64%	66%	55%	49%	47%	90%	84%	74%	69%	72%

Notes:

© 2024 Yang Z et al. *JAMA Network Open.*

(1) Explanations for the LTSS benefits (source: https://www.hhs.gov/guidance/document/reinterpretation-primarily-health-related-supplemental-benefits)

   a. Therapeutic Massage/Medically-Approved Non-Opioid Pain Management: Medically-approved non-opioid pain treatment alternatives, including therapeutic massage furnished by a state licensed massage therapist. "Massage" should not be singled out as a particular aspect of other coverage (e.g., chiropractic care or occupational therapy) and must be ordered by a physician or medical professional in order to be considered primarily health related and not primarily for the comfort or relaxation of the enrollee. The non-opioid pain management item or service must treat or ameliorate the impact of an injury or illness (e.g., pain, stiffness, loss of range of motion).
   b. Adult Day Care Services: Services provided outside the home such as assistance with ADLs/IADLs, education to support performance of ADLs/IADLs, physical maintenance/rehabilitation activities, and social work services targeted to ameliorate the functional/psychological impact of injuries or health conditions, or reduce avoidable emergency and healthcare utilization. Recreational or social activities or meals that are ancillary to primarily health related services and items may also be provided but the primary purpose of adult day care services must be health related and provided by staff whose qualifications and/or supervision meet state licensing requirements. Transportation to and from the adult day care facility may be provided and should be included in PBP B10b.
   c. Home-Based Palliative Care: Home-based palliative care services to diminish symptoms of terminally ill members with a life expectancy of greater than six months not covered by Medicare (e.g., palliative nursing and social work services in the home not covered by Medicare Part A). Medicare covers hospice care if a doctor and/or the hospice medical director certify the patient is terminally ill and has six months or less to live.
   d. In-Home Support Services: In-home support services to assist individuals with disabilities and/or medical conditions in performing ADLs and IADLs within the home to compensate for physical impairments, ameliorate the functional/psychological impact of injuries or health conditions, or reduce avoidable emergency and healthcare utilization. Services must be provided by individuals licensed by the state to provide personal care services, or in a manner that is otherwise consistent with state requirements.
   e. Support for Caregivers of Enrollees: Respite care provided through a personal care attendant or the provision of short-term institutional-based care, as appropriate, to ameliorate the enrollees' injuries or health conditions, or reduce the enrollees' avoidable emergency and healthcare utilization. Respite care should be for short periods of time (e.g., a few hours each week, a two week period, a four week period) and may include services such as counseling and training courses for caregivers of enrollees.

(2) Explanations for the SDOH benefits (source: https://www.hhs.gov/guidance/document/implementing-supplemental-benefits-chronically-ill-enrollees)

   a. General Supports for Living: General supports for living such as housing may be provided to enrollees if the benefit has a reasonable expectation of improving or maintaining the health or overall function of the enrollee. General supports for living may be provided for a limited or extended duration as determined by the plan. The benefit may include plan-sponsored housing

© 2024 Yang Z et al. *JAMA Network Open.*

consultations and/or subsidies for rent or assisted living communities. Plans may also include subsidies for utilities such as gas, electric, and water as part of the benefit. These benefits should be entered in Section B19b/13i of the PBP under “Transitional Supports.”

b. Food and Produce: Food and produce to assist enrollees in meeting nutritional needs. Plans may include items such as (but not limited to) produce, frozen foods, and canned goods. Tobacco and alcohol are not permitted.
c. Meals (beyond limited basis): Existing guidance in Chapter 4 of the Medicare Managed Care Manual provides that meals are a primarily health related benefit (PBP category B13c) in limited situations: when provided to enrollees for a limited period immediately following surgery, or an inpatient hospitalization, or for a limited period due to a chronic illness. In those situations, a meals supplemental benefit is permissible if the meals are: 1) needed due to an illness; 2) consistent with established medical treatment of the illness; and 3) offered for a short duration. Meals may be offered beyond a limited basis as a non-primarily health related benefit (PBP category B19b/13i) to enrollees. Meals may be home-delivered and/or offered in a congregate setting.
d. Pest Control: Pest eradication services that are necessary to ensure the health, welfare, and safety of the enrollee. Services may include pest control treatment(s) or products that may assist the enrollee in the pest eradication (e.g., traps, pest control sprays, cleaning supplies).
e. Transportation for Non-Medical Needs: Transportation to obtain non-medical items and services, such as for grocery shopping, banking, and transportation related to any other non-primarily health related benefit, is a non-primarily health related benefit. Such transportation may be reimbursed, arranged, or directly provided by an MA plan as a non-primarily health related benefit.
f. Indoor Air Quality Equipment and Services: Equipment and services to improve indoor air quality, such as temporary or portable air conditioning units, humidifiers, dehumidifiers, High Efficiency Particulate Air filters, and carpet cleaning. Plans may also include installation and servicing of equipment as part of the benefit.
g. Social Needs Benefits: Access to community or plan-sponsored programs and events to address enrollee social needs, such as non-fitness club memberships, community or social clubs, park passes, and access to companion care, marital counseling, family counseling, classes for enrollees with primary caregiving responsibilities for a child, or programs or events to address enrollee isolation and improve emotional and/or cognitive function.
h. Complementary Therapies: Complementary therapies offered alongside traditional medical treatment. Complementary therapies must be provided by practitioners who are licensed or certified, as applicable, in the state in which they practice and are furnishing services within the scope of practice defined by their licensing or certifying state. Alternative therapies that are considered primarily health related may be offered by an MA plan as a supplemental benefit in PBP category B14c. [Complementary therapy includes acupuncture, dietary supplements, massage therapy, hypnosis, and meditation. https://www.cancer.gov/publications/dictionaries/cancer-terms/def/complementary-therapy]
i. Services Supporting Self-Direction: Services supporting self-direction allow enrollees to have the responsibility for managing all aspects of healthcare delivery in a person-centered planning process; while such services are a non-primarily health related benefit, they may have a reasonable expectation of improving or maintaining the health or overall function of the enrollee. Plans

© 2024 Yang Z et al. *JAMA Network Open.*

may provide services to assist in the establishment of decision-making authority for healthcare needs (e.g., power of attorney for health services) and/or may provide education such as financial literacy classes, technology education, and language classes. Interpreter services may also be provided to enrollees to facilitate encounters with healthcare providers. Plans may not include expenses for funerals as a covered benefit. Primarily health related education (e.g., Health Education, Medical Nutrition Therapy) that is consistent with existing guidance (see Chapter 4, section 30.3) for primarily health related supplemental benefits may be offered by an MA Plan as a supplemental benefit in PBP category B14c.

j. Structural Home Modifications: Structural modifications to the home that may assist in the enrollee's overall function, health, or mobility are permitted if those items and services have a reasonable expectation of improving or maintaining the health or overall function of the enrollee (e.g., widening of hallways or doorways, permanent mobility ramps, easy use doorknobs and faucets).

© 2024 Yang Z et al. *JAMA Network Open.*

eTable 2. Percentage of MA plans offering LTSS or SDOH benefits, 2020-2024

	D-SNPs					Non–D-SNPs				
Year	2020	2021	2022	2023	2024	2020	2021	2022	2023	2024
N of enrollees	483	528	636	723	786	3,207	3,627	3,927	4,115	4,143
LTSS benefits:										
1 Therapeutic Massage	7%	6%	9%	11%	10%	4%	4%	5%	5%	5%
2 Adult Day Health Services	4%	5%	8%	9%	7%	1%	2%	2%	2%	2%
3 Home-Based Palliative Care	1%	1%	1%	1%	1%	2%	3%	3%	3%	4%
4 In-Home Support Services	15%	22%	34%	42%	28%	4%	6%	12%	16%	10%
5 Support for Caregivers of Enrollees	11%	2%	12%	16%	13%	2%	2%	5%	7%	7%
**Any LTSS benefits**	27%	29%	37%	46%	32%	9%	14%	18%	22%	18%
SDOH benefits:										
1 General Supports for Living	0%	4%	17%	29%	58%	0%	1%	3%	6%	7%
2 Food and Produce	4%	18%	50%	53%	68%	2%	4%	9%	12%	13%
3 Meals (beyond limited basis)	1%	8%	15%	13%	9%	1%	7%	6%	5%	4%
4 Pest Control	4%	9%	16%	14%	12%	2%	3%	4%	4%	3%
5 Transportation for Non-Medical Needs	2%	10%	21%	29%	34%	1%	2%	4%	5%	5%
6 Indoor Air Quality Equipment and Services	0%	5%	9%	10%	7%	0%	2%	2%	3%	2%
7 Social Needs Benefit	1%	6%	10%	16%	8%	1%	4%	4%	4%	3%
8 Complementary Therapies	0%	0%	7%	8%	7%	0%	0%	2%	2%	2%
9 Services Supporting Self-Direction	1%	4%	7%	9%	9%	0%	1%	2%	2%	1%
10 Structural Home Modifications	0%	0%	3%	3%	2%	0%	0%	1%	1%	0%
11 Other	4%	6%	17%	23%	26%	1%	4%	6%	5%	6%
**Any SDOH benefits**	7%	30%	57%	64%	70%	3%	12%	15%	16%	17%
Overall summary:										
Any LTSS or SDOH benefits	29%	43%	66%	72%	75%	10%	20%	26%	28%	28%
Any LTSS but no SDOH benefits	22%	13%	9%	8%	5%	7%	8%	11%	12%	11%
Any SDOH but no LTSS benefits	2%	14%	29%	26%	44%	1%	6%	7%	7%	10%
Both LTSS and SDOH benefits	5%	16%	28%	38%	26%	3%	6%	7%	9%	7%
No LTSS or SDOH benefits	71%	57%	34%	28%	25%	90%	80%	74%	72%	72%

Notes: See those under eTable 1.

© 2024 Yang Z et al. *JAMA Network Open.*

eTable 3. Association between area characteristics and county-level percentage of MA plans offering LTSS or SDOH benefits, 2024

		D-SNPs	
		LTSS	SDOH
	N of Counties = 2,713	Coeff. (95% CI)	Coeff. (95% CI)
**County MA penetration percentage (per 10 pp change)**	Mean (SD) 46.4 (13.8)	4.5 (3.8, 5.2)	4.1 (3.5, 4.7)
**County urban (vs. rural) status**	N (%) 1,294 (47.7%)	0.2 (-1.6, 1.9)	9.5 (8.0, 11.0)
**County Social Vulnerability Index (per 10 pp change)**	Mean (SD) 52.5 (28.4)	0.9 (0.5, 1.2)	0.1 (-0.1, 0.4)
**County Fully Integrated D-SNP enrollment percentage (per 10 pp change)**	Mean (SD) 7.4 (21.8)	1.7 (1.1, 2.2)	2.9 (2.5, 3.3)
**Approved state Medicaid HCBS waivers for individuals ages 65+ or with disabilities (vs. not)**	N (%) 2,251 (83.0%)	2.1 (0.1, 4.1)	-1.8 (-3.7, 0.2)

		Non–D-SNPs	
		LTSS	SDOH
	N of Counties = 3,047	Coeff. (95% CI)	Coeff. (95% CI)
**County MA penetration percentage (per 10 pp change)**	Mean (SD) 44.2 (15.1)	0.8 (0.5, 1.1)	0.0 (-0.3, 0.3)
**County urban (vs. rural) status**	N (%) 1,378 (45.2%)	3.4 (2.7, 4.1)	2.5 (1.8, 3.1)
**County Social Vulnerability Index (per 10 pp change)**	Mean (SD) 50.2 (28.7)	-0.1 (-0.3, 0.0)	0.2 (0.1, 0.3)
**Approved state Medicaid HCBS waivers for individuals ages 65+ or with disabilities (vs. not)**	N (%) 2,552 (83.8%)	5.0 (4.3, 5.7)	4.8 (4.2, 5.4)

Note: This table presents the results of multivariable linear regression models examining the association between county characteristics and plan offerings in 2024, weighted by county-level numbers of D-SNPs or non–D-SNPs, with robust standard errors.

© 2024 Yang Z et al. *JAMA Network Open.*
