# Supplementary material for: County-Level Enrollment in Medicare Advantage Plans Offering Expanded Supplemental Benefits
Source: JAMA Netw Open. 2024 Sep 17;7(9):e2433972. doi: 10.1001/jamanetworkopen.2024.33972 (PMC11409149; doi:10.1001/jamanetworkopen.2024.33972)
Supplement: Supplement 2. — Data Sharing Statement [file jamanetwopen-e2433972-s002.pdf]

Data Sharing Statement

Yang. County-Level Enrollment in Medicare Advantage Plans Offering Expanded Supplemental Benefits. *JAMA Netw Open*. Published September 17, 2024. doi:10.1001/jamanetworkopen.2024.33972

Data
Data available: Yes
Data types: Data (not involving human participants), Data dictionary
How to access data: zyang15@mgh.harvard.edu
When available: With publication

Supporting Documents
Document types: Statistical/analytic code
How to access documents: zyang15@mgh.harvard.edu
When available: With publication

Additional Information
Who can access the data: Anyone requesting the data.
Types of analyses: For any purpose.
Mechanisms of data availability: With investigator support.
